# Photo-Cleavable Peptide-Poly(Ethylene Glycol) Conjugate Surfaces for Light-Guided Control of Cell Adhesion

**DOI:** 10.3390/mi11080762

**Published:** 2020-08-08

**Authors:** Satoshi Yamaguchi, Yumi Takasaki, Shinya Yamahira, Teruyuki Nagamune

**Affiliations:** 1 Research Center for Advanced Science and Technology, The University of Tokyo, 4-6-1 Komaba, Meguro-ku, Tokyo 153-8904, Japan; 2 Precursory Research for Embryonic Science and Technology (PRESTO), Japan Science and Technology Agency (JST), 4-1-8 Hon-cho, Kawaguchi, Saitama 351-0198, Japan; 3 Department of Bioengineering, Graduate School of Engineering, The University of Tokyo, 7-3-1 Hongo, Bunkyo-ku, Tokyo 113-8656, Japan; 4 Center for Medical Sciences, St. Luke’s International University, 3-6-2 Tsukiji, Chuo-ku, Tokyo 104-0045, Japan

**Keywords:** cell micropatterning, photo-responsive material, cell adhesion, RGD peptide, PEG, microdevices

## Abstract

Photo-responsive cell attachment surfaces can simplify patterning and recovery of cells in microdevices for medicinal and pharmaceutical research. We developed a photo-responsive surface for controlling the attachment and release of adherent cells on a substrate under light-guidance. The surface comprises a poly(ethylene glycol) (PEG)-based photocleavable material that can conjugate with cell-adhesive peptides. Surface-bound peptides were released by photocleavage in the light-exposed region, where the cell attachment was subsequently suppressed by the exposed PEG. Simultaneously, cells selectively adhered to the peptide surface at the unexposed microscale region. After culture, the adhered and spread cells were released by exposure to a light with nontoxic dose level. Thus, the present surface can easily create both cell-adhesive and non-cell-adhesive regions on the substrate by single irradiation of the light pattern, and the adhered cells were selectively released from the light-exposed region on the cell micropattern without damage. This study shows that the photo-responsive surface can serve as a facile platform for the remote-control of patterning and recovery of adherent cells in microdevices.

## 1. Introduction

Microdevices for cell analysis and cell manipulation are being developed for various fields, from fundamental cell biology research to regenerative medicine [1]. Advantageously, microdevices require only a small amount of a cell sample and expensive analytical reagents. In addition, cells can be highly integrated with the devices by precise cell patterning, achieving image-based analysis of a large numbers of cells. In particular, single-cell arrays have recently received attention as an image-based, high-throughput cell sorting technology [2,3,4]. Moreover, precise cell patterning could produce in vivo-like cell-cell interactions [5] and artificial tissues [6] on a microdevice for cell diagnosis and organ-on-chip technologies, respectively. Thus, cell micropatterning is now a key technology for cell-analyzing microdevices. In recent years, there has been demand for a method to selectively recover target cells from cell micropatterns and further use the cells for analysis and engineering [2,3,4,5]. Therefore, a substrate surface that supports both precise patterning and selective release of cells is a promising tool for the development of simple and high-throughput microdevices for cell analysis and sorting.

Stimuli-responsive surfaces that respond to heat, voltage, and light have been developed for the non-invasive remote control of cell attachment to a substrate [7]. Photo-responsive surfaces are the most promising for controlling a large number of cells on microdevices because the spatial resolution of light is very high, enabling precise manipulation even at a single-cell level and light can be readily and quickly applied anywhere, even in closed spaces, if the materials are transparent. Therefore, numerous photo-responsive surfaces have been developed; however, most have been applied to only either cell patterning [8,9] or cell recovery [10,11,12]. Several photo-responsive surfaces have been reported to have light-induced control of both cell attachment and detachment [13,14,15,16,17,18]. Among these reports, some surfaces were produced using spiropyran derivatives, which are difficult to synthesize and are unstable against heat [13,14,15], or the surfaces required elaborate fabrication technologies [16] or exposure to ultraviolet light with a short wavelength [18]. Therefore, development of materials for photo-responsive surfaces that achieves both light-guided patterning and recovery of living cells is very important for a variety of practical uses. We previously reported a photocleavable poly(ethylene glycol) (PEG)-lipid as a simple material for such a photo-responsive surface [19,20,21,22]. On the material-coated surface, cells were selectively attached to the non-light-exposed region through interactions between the lipid moiety of the material and the cellular lipid bilayer membrane. A wide variety of cells can be patterned by light, regardless of their adhesiveness. Furthermore, the attached non-adherent cells can be released by exposure to light via photo-induced detachment of the lipid moiety [19,22]. However, on this surface, the adherent cells could not be photo-released because the cells were attached to the basement coating through biological adsorption after culture. In the adhered and spread state, most of the cells exhibit their native phenotype. To assay the native phenotype of adhered cells, a substrate surface was coated with collagen as a scaffold for cellular adhesion, followed by modification of the collagen coating with PEG-lipid at low density [20]. However, this photo-cleavable PEG-lipid surface cannot be used in light-guided selective cell recovery after image-based phenotype analysis of adherent cells.

**Figure 1:**
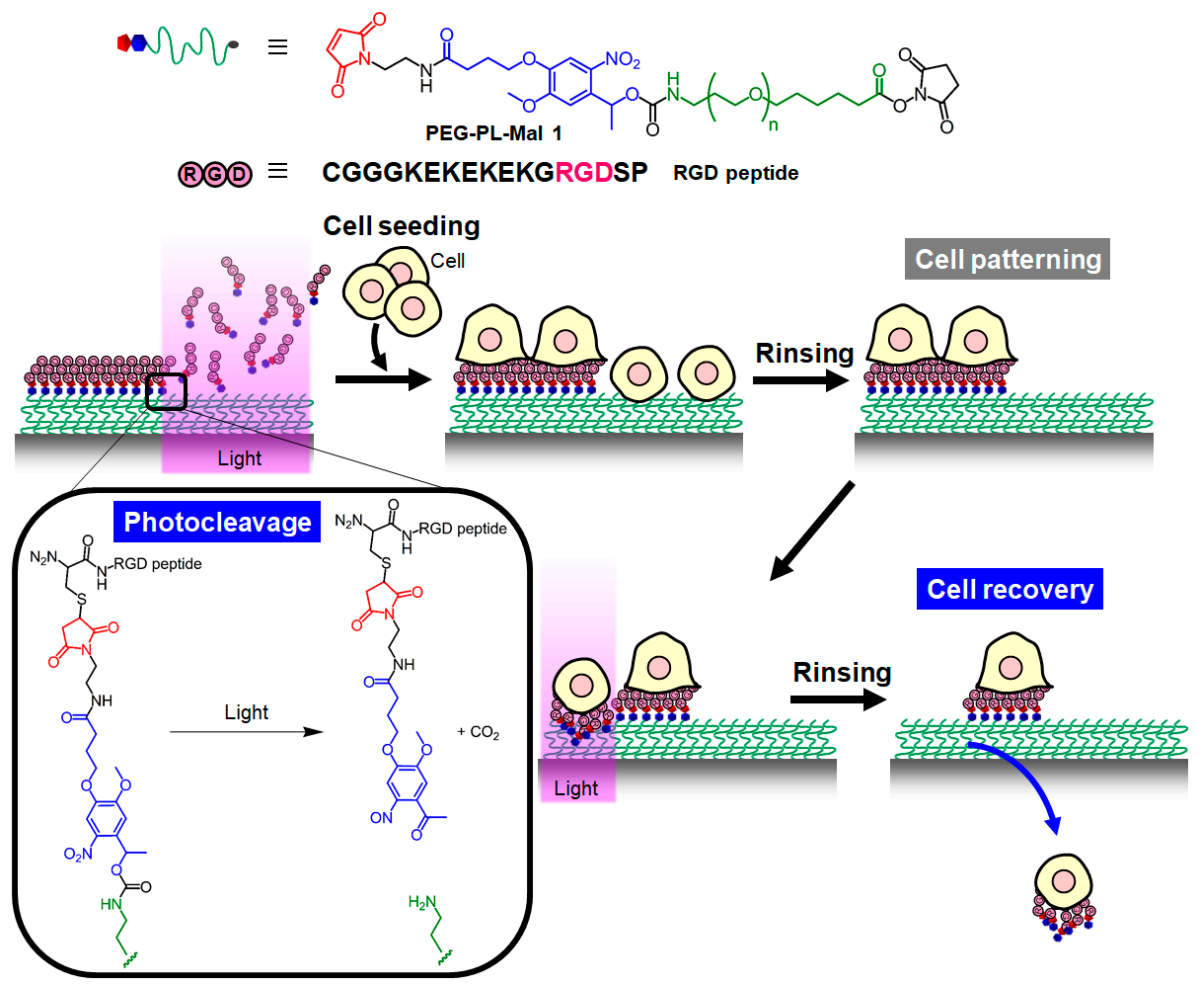
Schematic illustrations of light-guided cell patterning and recovery on a photocleavable RGD-PEG surface. The chemical structure of PEG-PL-Mal 1 and the amino acid sequence of the RGD peptide are shown (**top**) with the reaction scheme of the photocleavage on the surface (**left bottom**).

In this study, we developed a photocleavable material-based surface for light-guide pattering and recovery of adherent cells. Based on the simple molecular design of photo-cleavable PEG-lipid, a photocleavable substrate-coating material was synthesized by inserting a photolabile linker between the non-cell-adhesive PEG and a reactive group for tethering the cell-adhesive molecule. The reactive group bound to a peptide including the arginine-glycine-aspartic acid (RGD) motif as the cell adhesion molecule, leading to a photocleavable RGD-PEG surface. The RGD motif binds to integrins on the surface of adherent cells, which leads to cell adhesion and spreading [23], and is used for micropatterning of adherent cells [24]. On the RGD-PEG surface, the RGD peptide is designed to be released from the surface through a photocleavage reaction at the region exposed to light, where cell adhesion is inhibited. Simultaneously, cells are selectively adhered to the unexposed region, achieving facile light-guided cell patterning. Furthermore, the adhered cells are readily released from the desired region of the cell micropattern by exposure to non-cytotoxic light. Both the cell attachment and release on the surface in response to light were examined, and light-guided patterning and recovery of cells were conducted to show the usability of the surface (Figure 1).

## 2. Materials and Methods

### 2.1. Chemicals and Materials

4-[4-(1-Hydroxyethyl)-2-methoxy-5-nitrophenoxy]butyric acid (the photolabile linker) was purchased from Sigma-Aldrich (St. Louis, MO, USA). N-Hydroxysuccinimide (NHS), N-(2-Aminoethyl) maleimide hydrochloride, 1-(3-dimethylaminopropyl)-3-ethylcarbodiimide hydrochloride (WSC), chloroformic acid 4-nitrophenyl ester, triethylamine (TEA), N,N-diisopropylethylamine (DIPEA), mercaptoethanol and Trypan Blue were purchased from Tokyo Chemical, Inc. (Tokyo, Japan). Sunbright PA050HC (NH2-PEG5000-COOH) was purchased from NOF Corporation (Tokyo, Japan). 2-Aminopropyl triethoxysilane (APTES) was purchased from Shin-Etsu Silicone Co., Ltd. (Tokyo, Japan). Glass slides (35 mm × 25 mm, thickness 1.0–1.2 mm) were purchased from Matsunami Glass Ind. Ltd. (Tokyo, Japan). RGD peptide (CGGGKEKEKEKGRGDSP) [24] was purchased from Toray Research Center, Inc. (Otsu, Japan). Dulbecco’s modified Eagle medium (DMEM) was purchased from Nissui Seiyaku Co. Ltd. (Tokyo, Japan). Fetal bovine serum (FBS) was purchased from Biowest (Nuaillé, France). Calcein-AM was purchased from Dojindo Laboratories (Kumamoto, Japan). Penicillin/streptomycin was purchased from Nacalai Tesque Inc. (Kyoto, Japan).

### 2.2. Synthesis of the Photocleavable Substrate-Coating Material

**Scheme 1:**
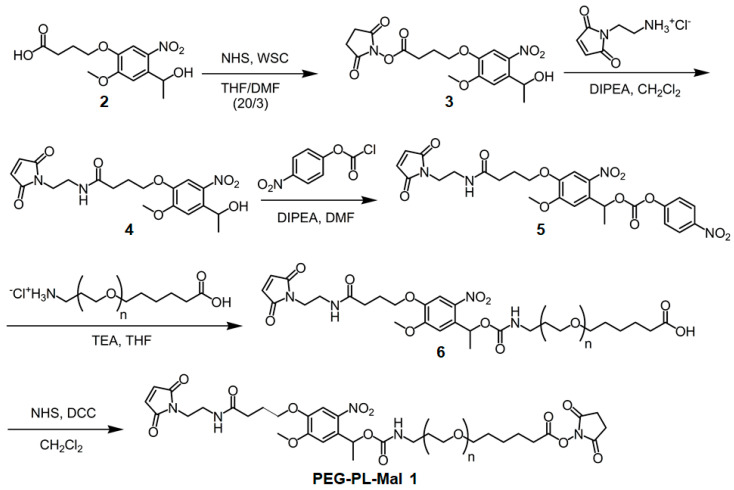
Synthetic scheme of PEG-PL-Mal 1.

A substrate-coating reagent, PEG-PL-Mal **1**, was synthesized in five simple steps from a commercially available starting compound **2** (Scheme 1). The details of the synthetic reactions are shown in the Supporting Information. The product was identified by ^1^H-NMR spectroscopy and photocleavage of PEG-PL-Mal 1 was confirmed by ^1^H-NMR spectroscopy (Figure S1).

### 2.3. Fabrication of the Substrate Surface

Introduction of the amine moiety to the substrate surface was conducted by amino-silanization with a silane coupling agent (APTES) [25]. A glass slide was washed 12 times with sterilized water (MilliQ water, Millipore Inc., MA, USA) and then immersed in a 0.1 M NaOH aqueous solution at 90 °C for 1 h. After washing with MilliQ water and drying, the glass slide was treated with an APTES solution (1% *v*/*v*, in a 0.1% acetic acid aqueous solution) at 90 °C for 1 h, followed by washing with MilliQ water.

The surface of the glass slide was then treated with a PEG-PL-Mal **1** solution (2 mM, in anhydrous dimethyl sulfoxide (DMSO)) at room temperature for 2 h. After washing the surface with Dulbecco’s phosphate-buffered saline (PBS) three times, the surface was treated with a solution of RGD peptide (50 mM, in PBS) at room temperature for 30 min. After washing with PBS, the surface was treated with a mercaptoethanol solution (0.2% *v*/*v*, in PBS) at room temperature for 30 min to quench the remaining maleimide moieties, resulting in a photocleavable RGD-PEG surface.

### 2.4. Photo-Responsive Cell Attachment and Release

Human cervical carcinoma cells (HeLa cells) were purchased from the RIKEN BioResource Research Center (Tsukuba, Japan). HeLa cells were cultured in DMEM supplemented with 10% (*v*/*v*) FBS and 0.5% penicillin/streptomycin at 37 °C under 5% CO_2_. For the cell attachment experiment, cells were harvested by treatment with 0.25% trypsin/EDTA solution. The cell pellet was suspended in serum-free DMEM at 5.0 × 10^5^ cells/mL.

For photo-responsive cell attachment, a part of the photocleavable RGD-PEG surface was exposed to light (4.0 J/cm^2^: 5.5 mW/cm^2^ × 727.2 s) with an ultraviolet (UV) irradiator (LAX-102, from Asahi Spectra Co., Ltd., Tokyo, Japan) equipped with a cylindrical lens through a bandpass filter (wavelength: 365 ± 5 nm). In this light-irradiation system, the superposition of nine circular lights from optical fibers emitted a uniform square light on the substrate surface. After washing the surface with PBS three times, the cell suspension was applied to the whole surface of the substrate, followed by incubation at 37 °C under 5% CO_2_ for 1 h. The cell-seeded surface was rinsed with a serum-free DMEM by pipetting to flush the non-adhered cells from the surface. The light-exposed region, the unexposed region, and their boundary region were observed with a fluorescent microscope (IX81, from Olympus Corp., Tokyo, Japan) before and after rinsing the surface.

For light-induced cell release, cells were first attached to the photocleavable RGD-PEG surface by incubation for 2 h. Next, a portion of the cell-adhered surface was exposed to light (4.0 J/cm^2^) and then the surface was rinsed. Similar to the cell attachment experiment, each region was microscopically observed before and after rinsing. The released cells in the medium were collected and then seeded onto a culture dish. As a control, the adhered cells on the RGD-PEG surface were collected by trypsin/EDTA treatment. The viability of collected cells was examined by staining with Trypan Blue and Calcein-AM.

### 2.5. Cell Micropatterning and Selective Release

The photocleavable RGD-PEG surface was prepared as described above. A simple photomask for the line pattern with the width of 400 μm was produced on a thin polyester film with an ordinary laser printer typically used for office work (ApeosPort-IV C3375, from Fuji Xerox Co. Ltd., Tokyo, Japan). The photomask film was attached to the substrate surface at the side opposite to the RGD-PEG surface. For cell patterning, the substrate surface was exposed to light through the photomask with the vertical line-pattern, followed by cell attachment on the surface as described above. For selective cell release, the direction of the line pattern on the photomask was changed to the horizontal direction. The surface was exposed to light through the photomask, followed by rinsing. The surfaces were microscopically observed before and after rinsing.

## 3. Results and Discussion

### 3.1. Photo-Responsive Cell Attachment

Cell attachment on the photocleavable RGD-PEG surface was examined at both the light-exposed and unexposed region. For the construction of the RGD-PEG surface, PEG-PL-Mal **1** was synthesized as a photo-responsive substrate-coating material. The surface was designed to display the maleimide moiety as the reactive group for tethering the RGD peptide via the photolabile linker on the PEG monolayer (Figure 1). The molecular design of RGD peptides with a cysteine residue at the N-terminus allows for simply conjugation with a maleimide moiety via a Michael reaction, and under exposure to light, the peptides detach from the PEG monolayer through the photocleavage of the linker (Figure 1, left bottom). In principle, at the unexposed region, a model HeLa cell attaches to the surface through adhesion via the RGD peptide, whereas the cell adhesion is inhibited at the light-exposed region because of the light-induced detachment of the RGD peptide (Figure 1).

**Figure 2:**
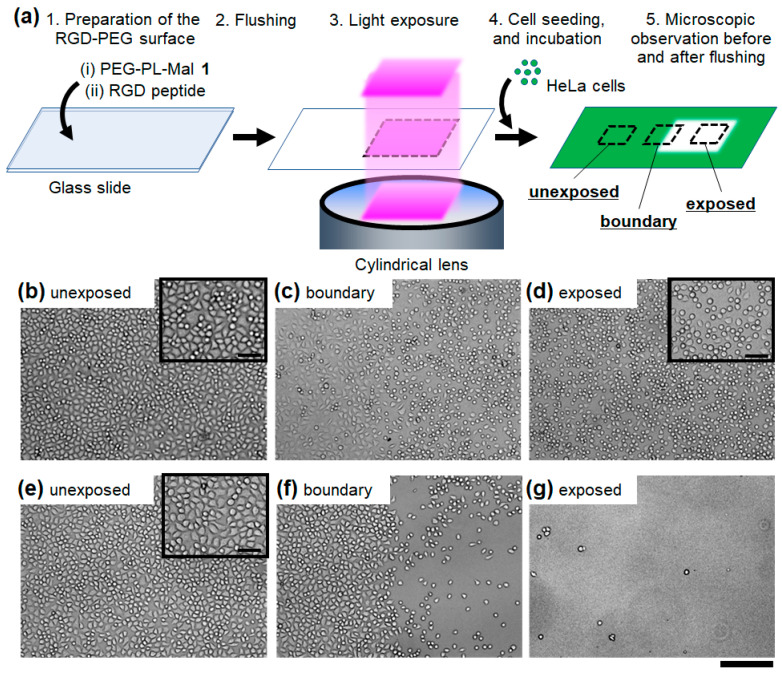
Cell adhesion on the photocleavable RGD-PEG surface exposed to light before cell seeding. (**a**) Schematic illustration of the photo-responsive cell attachment. HeLa cells on the surface were observed at the (**b**,**e**) unexposed region, (**c**,**f**) the boundary region between the unexposed (**left**) and exposed regions (**right**), and (**d**,**g**) the exposed region. The bright-field microscopic images were obtained (**b**–**d**) before and (**e**–**g**) after flushing the non-adhered cells from the surface. Light dose: 4.0 J/cm^2^. Scale bars: 200 μm. (**inset**) Magnified images. Scale bars: 50 μm.

To show proof of this principle, a part of the RGD-PEG surface was exposed to light by an optical-fiber-guided irradiation system, and then, cells were seeded to the whole surface (Figure 2a). After incubation for 1 h to induce cell adhesion to the surface, more than a half of the cells exhibited a spreading shape in the unexposed region, leading to full confluence (Figure 2b, inset). In the light-exposed region, most of the cells were round, with clear separation between cells because the cell size was smaller than those in the unexposed region (Figure 2d, inset). Moreover, at the boundary region, the cellular outline was more clearly visible in the light-exposed region (Figure 2c, right) compared with cells in the unexposed region (Figure 2c, left). This difference likely derives from the unattached cellular edge in the light-exposed region. These results strongly suggest that the cells adhered and spread on the surface only at the unexposed region. After flushing the non-adhered cells, nearly all of the cells remained at the unexposed region, whereas only a few cells were observed at the light-exposed region (Figure 2e–g). The non-specific detachment from the RGD-PEG surface not exposed to light was negligible; therefore, the cell adhesion through binding to the RGD peptide is sufficiently strong against flushing in the present experimental system. Additionally, the few attached cells in the light-exposed region indicates that the PEG layer without RGD peptides strongly inhibited non-specific cell adsorption to the substrate surface. Moreover, non-specific adsorbing cells were also sufficiently removed from the surface by flushing. The boundary does not show a sharp on-off difference in cell attachment between the exposed and unexposed surface because the light-irradiation system emitted blurred light at the edge of the uniform square light. Accordingly, there was a gradient in the light dose at the boundaries. Thus, photo-responsive attachment of adherent cells on the photocleavable RGD-PEG surface was confirmed.

### 3.2. Light-Induced Cell Release

**Figure 3:**
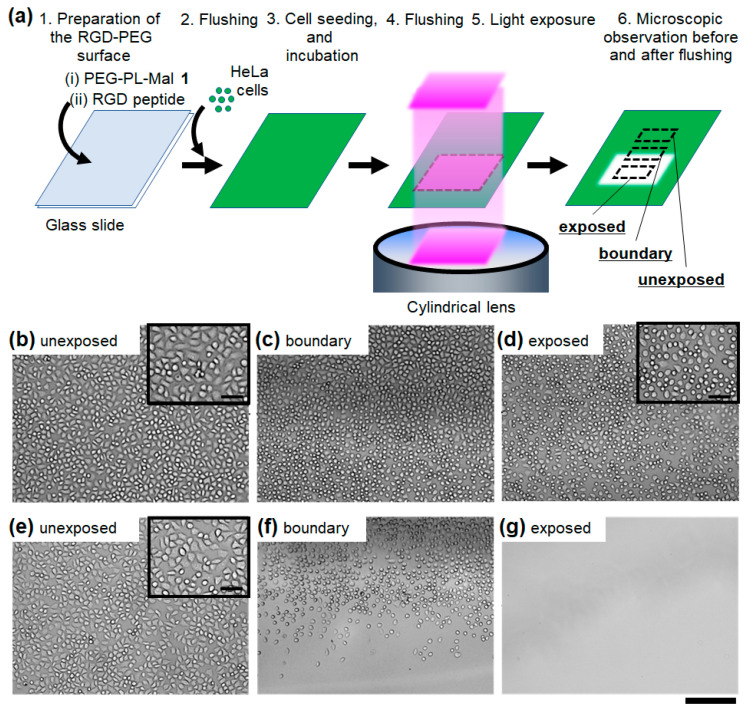
Cell release from the photocleavable RGD-PEG surface exposed to light after cell adhesion. (**a**) Schematic illustration of light-induced cell release. HeLa cells on the surface were observed at the (**b**,**e**) unexposed region, (**c**,**f**) the boundary region between the unexposed (**upper**) and exposed regions (**lower**) and (**d**,**g**) the exposed region. Bright-field microscopic images were obtained (**b**–**d**) before and (**e**–**g**) after flushing the detached cells from the surface. Light dose: 4.0 J/cm^2^. Scale bars: 200 μm. (**inset**) The enlarged images were observed. Scale bars: 50 μm.

The light-induced release of the adhered cells from the photocleavable RGD-PEG surface were evaluated. After adhesion of the HeLa cells, a part of the surface was exposed to light (Figure 3a). The light dose (365 nm, 4 J/cm^2^) is sufficient for photocleavage of the same photolabile linker that was used on a similar material-coated surface in our previous work without a significant impact on the cell viability [19]. After exposure to light, most of the cells were small and round in the light-exposed region (Figure 3c,d, lower), whereas nearly all of the cells remained spread in the unexposed region (Figure 3b,c, upper). In a control experiment, it was confirmed that no morphological change of HeLa cells adhered on plastic culture dishes was induced by the same light dose (Figure S2). This light-induced morphological change on the RGD-PEG surface suggests that the biological adhesion of the cells was inhibited by the loss of the traction with the substrate via the binding to the RGD peptide [24,26]. To release the detached cells from the substrate, the surface was rinsed with culture medium. After rinsing, no cells were observed in the light-exposed region (Figure 3f,g, lower). In the unexposed region, the adhered cells remained without changing the density or spread shape (Figure 3e,f, upper). Based on these results, the adhered cells can be released from the RGD-PEG surface in a light-induced manner.

The viability of the released cells was examined to show the usability of the photocleavable RGD-PEG surface as a tool for light-induced recovery of living cells. After rinsing the light-exposed surface, the released cells in the medium were collected. The viability of the collected cells was 97% (Figure S3) and the viability of cells collected with trypsin/EDTA treatment as a control was nearly 100%. This result shows the light-induced release caused negligible cytotoxicity. Consequently, the RGD-PEG surface supports the light-induced recovery of adhered living cells without cell damage.

### 3.3. Cell Micropatterning and Selective Recovery

**Figure 4:**
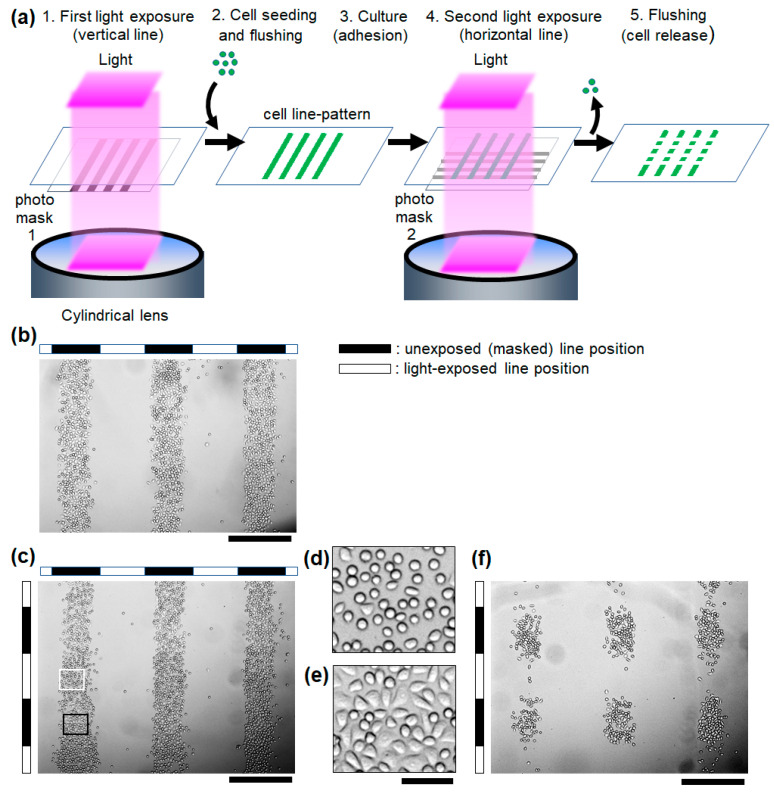
Light-guided micropatterning and release of adherent cells on the photocleavable RGD-PEG surface. (**a**) Schematic illustration of the cell patterning and release. (**b**) The microscopic image of the vertical line-pattern of HeLa cells after cell adhesion to the first exposed surface. (**c**) Cells immediately after the second exposure of the horizontal line-pattern of light. Scale bars: 500 μm. Magnified image of the cells in the (**d**) light-exposed (white square in (**c**)) and (**e**) unexposed regions on the horizontal line-pattern of the second exposure (black square in (**c**)). Scale bars: 50 μm. (**f**) The remaining cell pattern after the second light exposure and rinsing. Scale bars: 500 μm.

Light-guided cell micropatterning was demonstrated on the photocleavable RGD-PEG surface. First, the surface was exposed to light through a photomask with a vertical line pattern before cell seeding (Figure 4a). After cell adhesion and removal of the non-adhered cells using successive flushing, a vertical line pattern of the cells was obtained on the surface according to the mask pattern (Figure 4b). Most of the cells on the pattern were spread. The surface was then exposed to a horizontal line pattern of light (Figure 4c). In the light-exposed region, the cell shape became small and round (Figure 4d), and in the unexposed region, all of the cells remained spread (Figure 4e). After rinsing the surface, the light-exposed cells on the microscale pattern were selectively released from the surface in response to the line pattern of the second light exposure (Figure 4f). This result shows that the present photocleavable RGD-PEG surface can achieve both light-guided micropatterning of adherent cells and selective release of the adhered cells from the micropattern.

The present method simply achieved cell micropatterning by exposure of the modified surface to light patterns without complicated MEMS technologies, such as micro-molds, microwells, and microstructures, and expensive machines such as ink-jet printers. Compared with other cell micropatterning methods, such as microcontact printing [27] and ink-jet printing [28], no direct access to the substrate surface is required for placing ligands or cells. Accordingly, this surface can simplify the procedure for creating a cell micropattern in a closed microdevice such as microchannels. Thus, compared with other techniques, this method can construct micropatterns of adhered cells with similar or better resolution with less time and cost. The advantage is a result of the molecular design of the surface, which comprises a conjugate of the non-cell-adhesive PEG and a cell adhesive RGD peptide through photolabile linker (Figure 1). In a similar report in which an RGD peptide directly modified a substrate surface through a photolabile linker without PEG, cell patterning was not achieved even though the micropatterning of the peptide was reported [10]. This suggests that without the PEG layer, cells adhered to the surface regardless of the peptide pattern. Alternatively, in our previous work, a precise single-cell array of non-adhered cells was simply constructed using a similar PEG-based material that conjugated with a lipid chain as the cell-binding part [20,21,22]. From the results of this study and previous reports, suppression of non-specific cell adhesion to the substrate surface with PEG is considered a key to realizing precise control of cell patterning on the micrometer scale. Furthermore, though further study is needed, it is possible to make single-cell arrays of adherent cells using the present PEG-based material.

In this study, the cells adhered in the spread state could be selectively released from the developed surface in a light-induced manner. At that same time, since the light-exposed cells have lost their bond with the substrate surface, they were released from the surface by gentle pipetting (Figure 3). The easy detachment of the adhered cells suggests that the cells adhered to only the RGD peptides on the PEG layer as designed (Figure 1). If the attached cells had also adhered to the substrate surface by pushing aside the PEG layer, the cell detachment would have required a stronger rinse of the surface, probably leading to non-specific release even from the unexposed region. Therefore, severe inhibition of cell adhesion with the PEG layer is also critical for region-selective release of cells (Figure 4). Although some methods provide both cell attachment and detachment over a large region on the scale of millimeters [14,15,18], there are few reports in which both fine patterning and region-selective release on the micrometer scale can be achieved for adherent cells [16,17]. In these pioneering studies, cells were patterned on the substrate surface via photodegradable hydrogel scaffolds to firmly prevent direct adhesion to the substrate surface. However, in principle, such three-dimensional hydrogel surfaces require more light for cell release than a two-dimensional monolayer because more photolabile linker per unit area has to be cleaved for gel degradation. Specifically, a hydrogel-based surface with the same photolabile linker as this study was reported to require more light (12 J/cm^2^) for cell release [17] than the surface based on a PEG monolayer (4 J/cm^2^) presented in this study. Thus, the PEG-based molecular design may provide a more precise and less cytotoxic method given the reduced light exposure for light-guided cell recovery.

## 4. Conclusions

In this study, a photo-responsive surface for light-guided cell attachment and release was developed as a tool for remote control of cell adhesion. The surface was based on a new photocleavable PEG-based material that tethered an RGD peptide using a photolabile linker. On the RGD-PEG surface, a model adherent cell was selectively adhered to the unexposed region via binding to the RGD peptide on the PEG monolayer, which led to precise cell micropatterning. Moreover, the adhered cells in their spread state could be released without cell damage in a light-guided manner. The surface precisely achieved both light-guided micropatterning and recovery of the adhered cells due to inhibition of nonspecific cell adhesion with the PEG layer. The surface may be applied to the construction of single-cell arrays of adhered cells, followed by light-guided release of target cells after image-based analysis. Recently, image-based cell sorting technologies using a single-cell array have been used as a powerful tool for high-throughput isolation of rare cells from cell populations [2,3]. However, their application to adherent cells remains challenging because of the difficulty in selective release of adhered cells by conventional picking methods with a microcapillary tube. In principle, the proposed photo-responsive surface can assist selective picking of adhered single-cells using light-guided release to enable automated light-guided cell sorting systems for microfluidic devices.

## Supplementary Materials

The following are available online at https://www.mdpi.com/2072-666X/11/8/762/s1, Figure S1: ^1^H-NMR analysis of the photocleavage of PEG-PL-Mal **1**. (a) The scheme of the photocleavage reaction. (b) The ^1^H-NMR spectra of the PEG-PL-Mal **1** solution after exposure to light at 0 ~ 20.3 J/cm^2^. Figure S2: Microscopic images of the light-exposed HeLa cells on plastic culture dishes. (a) Before and (b) after exposure to light and (c) after rinsing. The yellow dotted line shows the boundary between the light-exposed region (upper) and unexposed region (lower). Scale bars: 500 μm. Figure S3: Microscopic images of the photo-released HeLa cells from the photocleavable RGD-PEG surface after Calcein-AM staining. (a) Bright-field image. (b) Green fluorescent image.
