# Supplementary material for: Photo-Cleavable Peptide-Poly(Ethylene Glycol) Conjugate Surfaces for Light-Guided Control of Cell Adhesion
Source: Micromachines (Basel). 2020 Aug 8;11(8):762. doi: 10.3390/mi11080762 (PMC7465029; doi:10.3390/mi11080762)
Supplement: Supplementary file 1 [file micromachines-11-00762-s001.pdf]

Supplementary Materials: Photo-Cleavable Peptide-Poly(Ethylene Glycol) Conjugate Surfaces for Light-Guided Control of Cell Adhesion

Satoshi Yamaguchi, Yumi Takasaki, Shinya Yamahira and Teruyuki Nagamune

1. General Procedures and Materials for Organic Synthesis

4-[4-(1-Hydroxyethyl)-2-methoxy-5-nitrophenoxy]butyric acid (the photolabile linker) was purchased from Sigma-Aldrich (St. Louis, MO). *N*-Hydroxysuccinimide (NHS), *N*-(2-aminoethyl) maleimide hydrochloride, 1-(3-dimethylaminopropyl)-3-ethylcarbodiimide hydrochloride (WSC), dicyclohexylcarbodiimide (DCC), chloroformic acid 4-nitrophenyl ester, triethylamine (TEA) and N,N-diisopropylethylamine (DIPEA) were from Tokyo Chemical, Inc. (Tokyo, Japan). Sunbright PA050HC (NH_2-PEG5000-COOH) was from NOF Corporation (Tokyo, Japan).

All other reagents were commercially available and used as supplied without further purification. Column chromatography was performed on a silica gel provided by Kanto Chemical Co. Inc. (60N spherical, 40–50 μm). NMR chemical shifts are reported in ppm downfield of tetramethylsilane using a residual solvent as an internal reference. NMR spectra were recorded with a FT-NMR spectrometer (JEOL; JNM-A500) using a NM-50TH5 probe (500 MHz).

2. Synthesis of PEG-PL-Mal 1

A substrate-coating reagent, PEG-PL-Mal **1** was synthesized in simple five steps from a commercially available starting compound **2** (Scheme 1). The details of the synthetic reactions were shown as follows:

Synthesis of 3.

4-[4-(1-Hydroxyethyl)-2-methoxy-5-nitrophenoxy]butyric acid (**2**) (150 mg, 0.50 mmol) and NHS (85.2 mg, 0.74 mmol) was dried up and then dissolved into a mixture of dry THF (2 mL) and dry DMF (0.3 mL) in Ar atmosphere. Then, WSC (140 mg, 0.73 mmol) was added to start reaction. After reacted for 1 day, the solvent was removed by evaporator to obtain crude product. The crude product was suspended in 5 mL of H_2O, and the yellow solid of **3** was separated by filtration, followed with drying in a vacuum. Yellow solid of **3** was obtained (181 mg, yield: 91 %).

^{1}H-NMR of one (500 MHz, CD_3OD) δ: 7.59 (s, 1H), 7.29 (s, 1H), 5.56 (q, 1H), 4.17 (t, 2H), 3.99 (s, 3H), 2.85 (dt, 6H), 2.30 (m, 2H), 1.56 (d, 3H).

Synthesis of 4.

Compound **3** (150 mg, 0.38 mmol) and *N*-(2-Aminoethyl) maleimide hydrochloride (80.5 mg, 0.45 mmol) were dried up and then dissolved into 4 ml of dry DCM in Ar atmosphere. Then, anhydrous DIPEA (250 μl, 1.40 mmol) were added to start reaction. After reacted for 6 h, the reaction mixture was concentrated by evaporator, and the product was purified with silica column chromatography (DCM/MeOH: 12/1). Yellow solid of **4** was obtained (101 mg, yield: 63 %).

^{1}H-NMR of one (500 MHz, CD_3OD) δ: 7.57 (s, 1H), 7.30 (s, 1H), 6.69 (s, 2H), 6.03 (bs, 1H), 5.56 (q, 1H), 4.09 (t, 2H), 3.98 (s, 3H), 3.69 (t, 2H), 3.47 (t, 2H), 2.37 (t, 2H), 2.16 (m, 2H), 1.56 (d, 3H).

Synthesis of 5.

Compound **4** (65.0 mg, 0.15 mmol) and 4-nitrophenyl chloroformate (54.5 mg, 0.27 mmol) were dried up and then dissolved into 2 ml of dry DMF in Ar atmosphere. Then, anhydrous DIPEA (257 μl, 1.44 mmol) were added to start reaction. After reacted for 5 h, the reaction mixture was

concentrated by evaporator, and the product was purified with silica column chromatography (AcOEt/Hexane: = 6/1). Yellow solid of **5** was obtained (44.5 mg, yield: 49 %).

^{1}H-NMR of one (500 MHz, CD_3OD) δ: 8.26 (d, 2H), 7.60 (s, 1H), 7.34 (d, 2H), 7.11 (s, 1H), 6.69 (s, 2H), 6.52 (q, 1H), 6.12 (bs, 1H), 4.10 (t, 2H), 4.01 (s, 3H), 3.70 (t, 2H), 3.46 (t, 2H), 2.37 (t, 2H), 2.17 (m, 2H), 1.78 (d, 3H).

Synthesis of 6.

Compound **5** (35.9 mg, 62 μmol) and Sunbright PA-050HC (126.0 mg, 25 μmol) were dried up and then dissolved into 3 ml of dry DCM in Ar atmosphere. Then, anhydrous TEA (210 μl, 1.5 mmol) were added to start reaction. After reacted for 5 h, the reaction mixture was concentrated by evaporator, and the product was precipitated by adding 50 mL of cold diethylether (-20 °C), followed by centrifugation at 10000 *g* for 10 min at -20 °C. The supernatant was removed by decantation, and the product was dried in a vacuum. White solid of **6** was obtained (123 mg, yield: 90 %)).

^{1}H-NMR of one (500 MHz, CD_3OD) 7.57 (s, 1H), 7.00 (s, 1H), 6.68 (s, 2H), 6.33 (q, 1H), 6.16 (bs, 1H), 5.60 (bs, 1H), 4.07 (t, 2H), 3.95 (s, 3H), 3.82 (t, 2H), 3.63-3.47 (bm, PEG), 3.25 (m, 2H), 2.34 (dt, 4H), 2.16 (m, 2H), 1.75 (m, 2H), 1.65-1.41 (bm, 9H).

Synthesis of PEG-PL-Mal (1).

Compound **6** (123 mg, 23 μmol) and NHS (5.40 mg, 47 μmol) were dried up and then dissolved into 2 ml of dry DCM in Ar atmosphere. Then, DCC (11.0 mg, 53 μmol) were added to start reaction. After reacted for 4 h, the reaction mixture was filtered, concentrated and then precipitated by adding 80 mL of diethyl ether, followed by centrifugation at 10000 *g* for 10 min at -10 °C. The supernatant was removed by decantation, and the product was dried in a vacuum. White solid of **1** was obtained (134 mg, yield: >99 %).

^{1}H-NMR of one (500 MHz, CD_3OD) 7.57 (s, 1H), 7.01 (s, 1H), 6.68 (s, 2H), 6.33 (q, 1H), 6.16 (bs, 1H), 5.60 (bs, 1H),4.08 (t, 2H), 3.95 (s, 3H), 3.82 (t, 2H), 3.63-3.47 (bm, PEG), 3.22 (m, 2H), 2.85 (bt, 4H), 2.61 (t, 2H), 2.37 (t, 2H), 2.15 (m, 2H), 1.77 (bm, 4H), 1.61 (bm, 5H), 1.48 (bm, 2H).

3. ^{1}H-NMR Analysis of the Photocleavage of PEG-PL-Mal 1

A solution of PEG-PL-Mal **1** (2 mM, in $CDCl_3$) was put into a glass vial and exposed to various doses of light (365 nm) with a xenon light source through a band-pass filter (± 5 nm; MAX-302, from Asahi Spectra Co. Ltd., Tokyo, Japan). Then, the ^{1}H-NMR spectra of the light-irradiated solutions were measured (Figure S1).

The chemical shifts of the proton x and y of PEG-PL-Mal **1** disappeared after exposure to light at 20.3 J/cm^2, and simultaneously, those of proton x′ and y′ of the photocleaved product appeared (Figure S1). Here, the perfect cleavage of high-concentrate PEG-PL-Mal **1** required a high dose of light. But, on the substrate, the required dose of light was extremely lower because the amount of the material on the surface is very small.

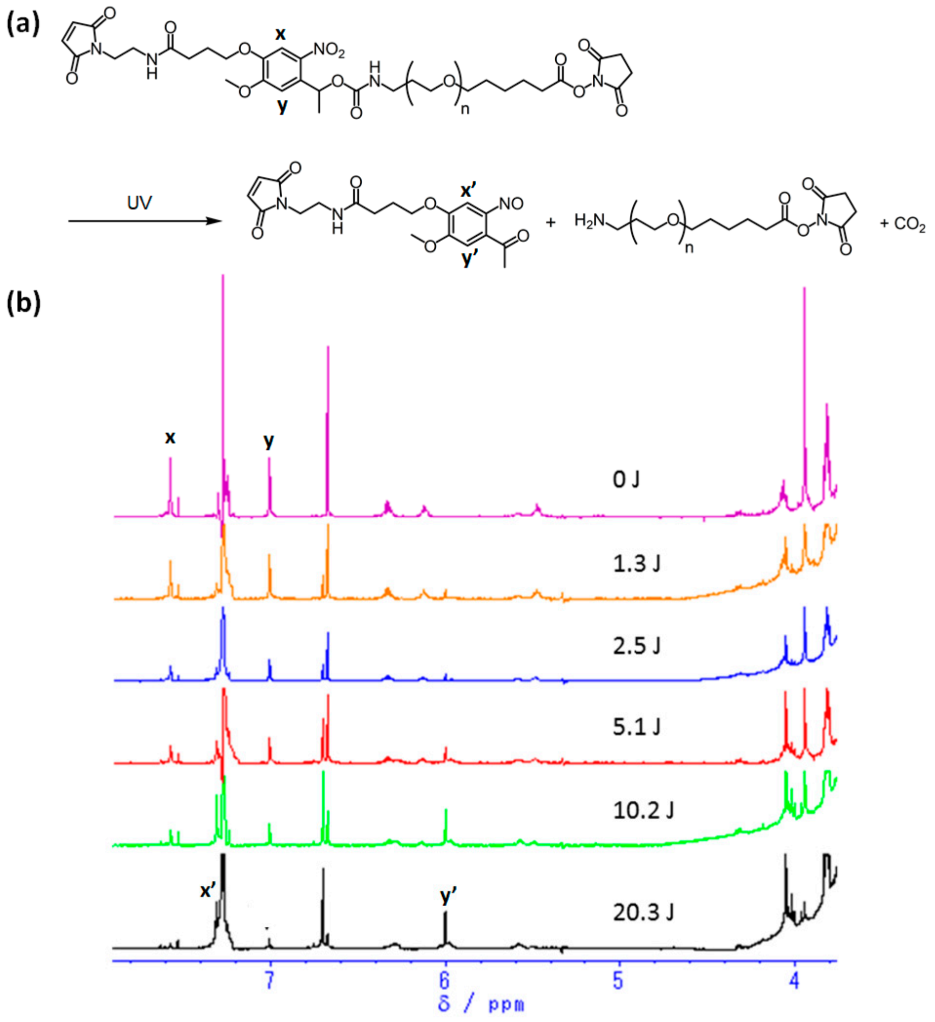

Figure S1. ^{1}H-NMR analysis of the photocleavage of PEG-PL-Mal **1**. (**a**) The scheme of the photocleavage reaction. (**b**) The ^{1}H-NMR spectra of the PEG-PL-Mal **1** solution after exposure to light at 0 ~ 20.3 J/cm^2.

4. Morphological observation of the light-exposed cells

Human cervical carcinoma (HeLa cell) was seeded on plastic culture plates and cultured as described at the Materials and Methods section 2.4. A part of the dish surface was exposed to light (4.0 J/cm^2: 5.5 mW/cm^2 × 727.2 sec) with an ultraviolet (UV) irradiator (LAX-102, from Asahi Spectra Co., Ltd., Tokyo, Japan) equipped with a cylindrical lens through a bandpass filter (wavelength: 365 ± 5 nm). Then, the dish surface was rinsed with serum-free Dulbecco's modified eagle's medium (DMEM) by pipetting to flush detached cells from the surface. The boundary region between the light-exposed and unexposed regions were observed with a fluorescent microscope (IX81, from Olympus Corp., Tokyo, Japan) before and after exposure to light and after rinsing.

Figure S2 shows the microscopic images of light-exposed HeLa cells in almost the same area before and after light exposure and after rinsing. No morphological change in the light-exposed region was observed before and after light exposure (Figure S2a and 2b, *upper*). The cell density in the light-exposed region did not change before and after rinsing (Figure S2b and 2c, *upper*).

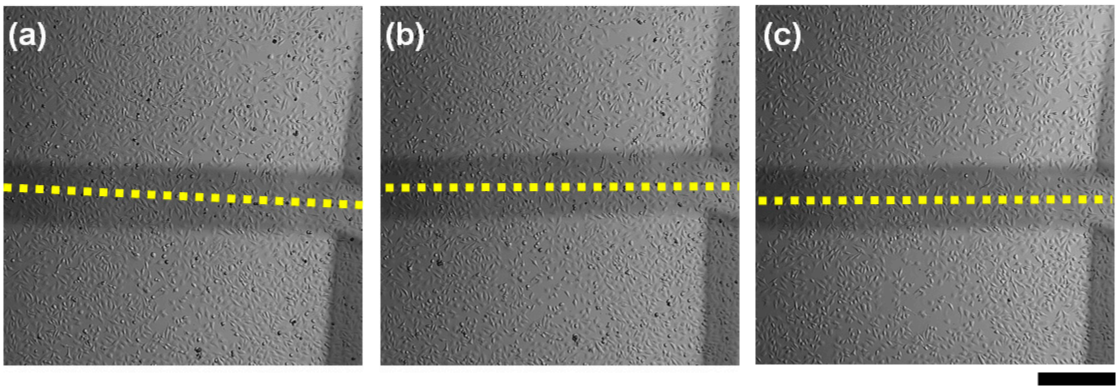

Figure S2. Microscopic images of the light-exposed HeLa cells on plastic culture dishes. (**a**) Before and (**b**) after exposure to light and (**c**) after rinsing. The yellow dotted line shows the boundary between the light-exposed region (*upper*) and unexposed region (*lower*). Scale bars: 500 μm.

5. Viability Assay of the Photo-Released Cells

The photocleavable RGD-PEG surface was prepared as described at the Materials and Methods section 2.3. Human cervical carcinoma (HeLa cell, 5.0 × 10^5 cells/mL) was seeded and attached on the surface by incubation for 2 hours as described at the Materials and Methods section 2.4. The cell-attached surface was rinsed with serum-free Dulbecco's modified eagle's medium (DMEM) by pipetting to flush weakly adsorbed cells from the surface. Then, a part of the cell-attached surface was exposed to light (4.0 J/cm^2) with an ultraviolet (UV) irradiator (LAX-102, from Asahi Spectra Co., Ltd., Tokyo, Japan) equipped with a cylindrical lens through a bandpass filter (wavelength: 365 ± 5 nm). The photo-released cells were collected with the medium. The remaining cells at the unirradiated region were harvested from the substrate as a control by the trpsin/EDTA treatment. A part of the collected cells was stained with Trypan Blue, and then, both the stained and non-stained cells were counted with a microscope. The viability was determined by dividing the number of the stained cells with that of the total cells. As a result, the viability of the photo-released and enzymatically released cells were 97 % and 100 %, respectively. Furthermore, another part of the photo-released cells was stained with Calcein-AM, followed with observation by fluorescence microscopy.

Figure S3 shows the microscopic images of the Calcein-AM-stained cells. Almost all cells were observed to emit the green fluorescence, indicating that almost all cells were alive as shown by Trypan Blue staining.

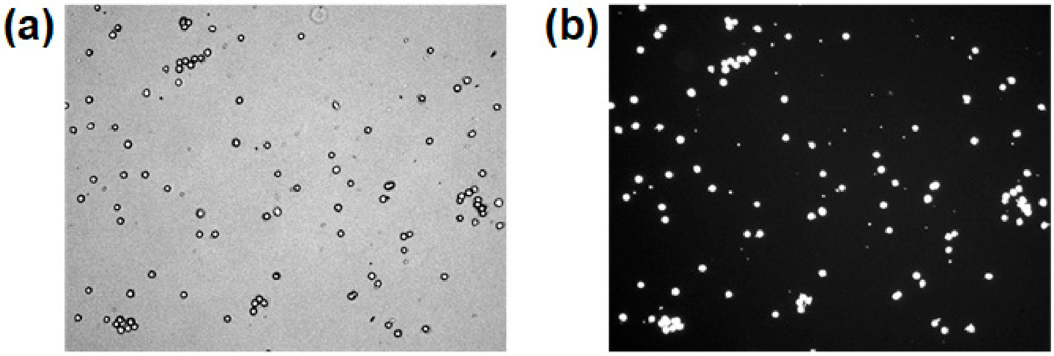

Figure S3. Microscopic images of the photo-released HeLa cells from the photocleavable RGD-PEG surface after Calcein-AM staining. (**a**) Bright-field image. (**b**) Green fluorescent image.
